# Blood Feeding and *Plasmodium* Infection Alters the miRNome of *Anopheles stephensi*

**DOI:** 10.1371/journal.pone.0098402

**Published:** 2014-05-27

**Authors:** Shanu Jain, Vandita Rana, Jatin Shrinet, Anil Sharma, Adak Tridibes, Sujatha Sunil, Raj K. Bhatnagar

**Affiliations:** 1 International Centre for Genetic Engineering and Biotechnology, New Delhi, India; 2 National Institute of Malaria Research, Dwarka, New Delhi, India; Centro de Pesquisas René Rachou, Brazil

## Abstract

Blood feeding is an integral process required for physiological functions and propagation of the malaria vector *Anopheles*. During blood feeding, presence of the malaria parasite, *Plasmodium* in the blood induces several host effector molecules including microRNAs which play important roles in the development and maturation of the parasite within the mosquito. The present study was undertaken to elucidate the dynamic expression of miRNAs during gonotrophic cycle and parasite development in *Anopheles stephensi*. Using next generation sequencing technology, we identified 126 miRNAs of which 17 were novel miRNAs. The miRNAs were further validated by northern hybridization and cloning. Blood feeding and parasitized blood feeding in the mosquitoes revealed regulation of 13 and 16 miRNAs respectively. Expression profiling of these miRNAs revealed that significant miRNAs were down-regulated upon parasitized blood feeding with a repertoire of miRNAs showing stage specific up-regulation. Expression profiles of significantly modulated miRNAs were further validated by real time PCR. Target prediction of regulated miRNAs revealed overlapping targeting by different miRNAs. These targets included several metabolic pathways including metabolic, redox homeostasis and protein processing machinery components. Our analysis revealed tight regulation of specific miRNAs post blood feeding and parasite infection in *An. stephensi*. Such regulated expression suggests possible role of these miRNAs during gonotrophic cycle in mosquito. Another set of miRNAs were also significantly regulated at 42 h and 5 days post infection indicating parasite stage-specific role of host miRNAs. This study will result in better understanding of the role of miRNAs during gonotrophic cycle and parasite development in mosquito and can probably facilitate in devising novel malaria control strategies at vector level.

## Introduction

Vector transmitted diseases are widespread in tropics and sub-tropics. Of several vector transmitted diseases, malaria accounts for more than 200 million cases with India contributing to almost 70% of malarial cases in South East Asia region [1]. Malaria was brought on the verge of eradication by the success of malaria eradication program by World Health Organisation in 1950s. But 1990s saw resurgence in malarial incidence due to insecticide resistance in vectors, drug resistance in parasites, changes in vector behaviour and lack of infrastructure to fight the disease. India has an intricate vector dynamics with six primary vectors of the 58 *Anopheles* species reported in the country [2]. Of these, *Anopheles stephensi* is considered as an important vector with its distribution throughout the Middle East and South Asia region.

*Plasmodium* passes through sexual and asexual stages of its development in mosquito and mammals respectively in order to complete its life cycle. Gametocytes transmitted to mammalian host by an infected mosquito develop into a motile ookinete which traverses through midgut epithelium and develop into an oocyst. Sporozoites burst out of oocyst, travel through hemolymph and invade salivary gland acinar cells. These sporozoites are then transmitted to a mammalian host upon next blood meal of mosquito. The several motile stages of parasite initiate an array of humoral and cellular immune responses in the mosquito. Activation of these pathways culminates in production of antimicrobial effector molecules, which function as either agonist or antagonist during maturation of *Plasmodium* parasite [3]. Previous studies have shown that blood feeding and *Plasmodium* infection leads to differential expression of genes in the mosquito [4], [5]. Among the various factors known to regulate gene expression, microRNAs (miRNAs) have emerged as the most important regulatory molecules. MiRNAs are 22 nucleotides (nt) non-protein coding small RNAs shown to play role in multiple biological processes including defense response of host against invading pathogen [6]. Primary miRNAs are transcribed by RNA polymerase II [7] and is processed off its flanking regions in nucleus by an RNAse III enzyme, Drosha [8] giving rise to precursor miRNAs (pre-miRNA). Pre-miRNA is then exported to the cytoplasm by exportin -5 RanGTP complex [9]–[10] where it is processed by Dicer, into 22 nt miRNA duplex [11]–[13]. One strand of miRNA duplex binds to 3 ' untranslated region (UTR) of target genes and regulates their expression post-transcriptionally. A single miRNA has been shown to regulate expression of thousands of mRNAs involved in diverse biological pathways [14]–[15].

Recent efforts towards control of spread of malaria have focussed on blocking *Plasmodium* cycle within the mosquito [16]–[17]. An increased understanding of vector-parasite interaction is essential to identify vector specific molecules that could be manipulated to block maturation of *Plasmodium*. Several studies have been conducted to understand role of miRNAs during growth and development in *Drosophila* [18]–[20]. Small RNA sequencing and computational methods have been successfully applied to identify putative miRNAs and their targets in *Drosophila* [21]–[24] and non-Drosophiloid insects. In comparison, scant information is available on differentially expressed miRNAs in mosquito at different stages of parasite maturation [6]. A recent study on *An. stephensi* has identified 27 miRNAs by direct cloning method. Further, they identified miRNAs implicated in mosquito reproduction and longevity [25].

Our present study was conducted to understand the role of *Anopheles* miRNAs during *Plasmodium* infection. For this purpose, we performed high throughput sequencing on *An. stephensi* mosquitoes after blood feed and at different time points of *Plasmodium* development in the vector. We identified 126 miRNAs of which 17 were novel miRNAs. Further analysis of these miRNAs revealed regulation of 13 miRNAs during blood feeding and 16 miRNAs upon *Plasmodium* infection. Additionally, we performed target prediction of these miRNA clusters to deduce their potential role. Our bio-informatic analysis predicted these regulated miRNAs to be playing roles in pathways directly affecting reproduction and parasite development in mosquito.

## Materials and Methods

### Ethics statement

Animal experiments were performed in accordance with National animal ethics guidelines of the Government of India after approval by Institutional Animal Ethics Committees of International Centre for Genetic Engineering & Biotechnology, New Delhi (Permit number: ICGEB/AH/2011/01/IR-8).

### Mosquito rearing and infection

*An. stephensi* from laboratory stock were reared under controlled conditions at 28+2°C, 70–75% humidity. Adult mosquitoes were fed with 2% sterile glucose solution and water soaked raisin. Stock solution of *Plasmodium vinckei petteri* 279 BY was thawed and injected into 5 weeks old BALB/C mice. When gametocytemia reached 0.05%, naive 4–5 days old female mosquitoes were fed on infected mice. Midguts of mosquitoes on 5^th^ day post infection (dpi) were dissected, stained in mercurochrome and observed for the presence of oocysts under microscope. Presence of oocysts confirmed infection of mosquitoes by *Plasmodium* parasite.

### Sample preparation

Samples were prepared from *An. stephensi* at five different conditions. Sugar fed adult female mosquitoes 5–6 days old (SF) were collected as control sample. Blood fed (BF 42 h and BF 5d) and parasite infected mosquitoes (iBF 42 h and iBF 5d) were collected at two time points, at 42 hours and five days post blood feeding. Whole body of all mosquito samples were stored in Trizol (Invitrogen) until RNA extraction. Total RNA enriched in small RNA population was extracted using miRNeasy kit (Qiagen) as per the manual's protocol. Quality and quantity of RNA was checked by using Agilent 2100 Bioanalyzer RNA Nano 6000 kit.

### Small RNA sequencing

Illumina Truseq small RNA libraries were made as per the manufacturer's instructions (Illumina Inc). 1 µg of total RNA was ligated with 3′ and 5′ adaptors followed by reverse transcription using RT primers. Following PCR amplification of the adaptor enriched fragments, amplified products were ran and small RNA population within length 140–160 base pairs (bps) were eluted from 6% TBE PAGE gel. Eluted product was precipitated using sodium acetate and ethanol and was dissolved in RNAase free water. These small RNA libraries were then sequenced using Illumina Genome Analyzer II.

### Computational analysis of small RNA sequencing data

Raw data generated post Deep sequencing was processed following an in-house pipeline described in detail [26] with slight modifications. Briefly, mature and pre-miRNA sequences of available eight insect species namely *Anopheles gambiae, Aedes aegypti, Culex, Drosophila melanogaster, Bombyx mori, Apis mellifera, Acyrthosiphon pisum, Tribolium castaneum* were downloaded from mirbase database v.19 [27]. To reduce data redundancy, 100% similar sequences were pooled together using CDHIT web tool [28]. All available non-coding RNA sequences (ncRNAs) and protein coding region of *Ae. aegypti, An. stephensi* and *An. gambiae* were downloaded using ncRNA database [29] and vectorbase [30] respectively. All downloaded sequences were indexed to create four different databases using Bowtie [31]. Data generated after sequencing all five libraries were analysed separately.

Reads derived from deep sequencing were trimmed and filtered to fetch sequences having length > = 18 bases (nt). In each library, same sequences were pooled together to generate expression files (fasta) with their read count and unique ID used for further analysis. These unique reads were aligned against mature miRNA sequence database using Bowtie with zero mismatches and taking other parameters as default.

For identification of novel miRNAs, unmatched sequences after known miRNA prediction were mapped to pre-miRNA sequences. Further, to remove non-coding RNAs, sequences left unmatched to pre-miRNAs were mapped to ncRNA database. Sequences that remained unmatched after this step were mapped to coding region of *Ae. aegypti, An. stephensi* and *An. gambiae* to filter out the sequences falling in coding regions. The final sets of unmatched sequences were then matched to *An.stephensi* genome. These matched sequences were subjected to novel miRNA prediction. 75 nt flanking region from both sides of genome sequence that matched with small RNA sequence was fetched as precursor sequence. These precursor sequences were folded to generate secondary structures and their folding energies were calculated using RNAfold [32] and RNAplot [32]. Precursor sequences forming hairpin loop structure with energy < = −20 KJ/mol and small RNA sequence lying in one arm of precursor were reported as novel Pre-miRNAs and mapping small RNA sequence as mature miRNAs.

To analyze relative abundance and expression profiling of miRNAs, tags per million of total RNA reads (TPM) for each miRNA in all five libraries were calculated. TPM was compared between the libraries to identify miRNAs differentially expressed post blood feeding and infection in mosquito.

### Expression profiling of miRNA by real time PCR

The Custom miRNA locked nucleic acid PCR primer sets (Exiqon) were designed using target miRNA sequences from closely related species. 10 ng of Total RNA was reverse transcribed into cDNA using universal cDNA synthesis kit (Exiqon). Real time PCR was set up with two biological replicates each in triplicates using SYBR green master mix (Exiqon) following manufacturer's instructions in ABI one step detection system. 5.8 s rRNA was used as an endogenous control for miRNA expression profiling. Expression levels were then calculated against SF as a calibrator using 2^−ΔΔC^_T_ method.

### Northern blot

Northern hybridizations were conducted using digoxigenin-labelled antisense miRCURY LNA probes (Exiqon). Total RNA enriched in small RNA population was extracted using mirVana miRNA isolation kit (Invitrogen) from female mosquitoes collected at 42 h post blood feeding. 10 µg of total RNA was loaded in a 15% denaturing polyacrylamide gels. Gels were stained with ethidium bromide for verification of RNA quality before transferring to a nylon membrane. Following l-ethyl-3-(3-dimethylaminopropyl) carbodiimide crosslinking, membranes were pre-hybridized in a rotating hybridization oven for 30 min at 37°C in hybridization buffer. 0.5 nM miRCURY LNA probe (Exiqon) was added to the same buffer at 37°C for overnight hybridization. All probes were designed against *Drosophila* miRNA sequences. The membranes were washed twice for 10 min each in a low stringent buffer (2X saline sodium citrate, 0. 1% sodium dodecyl sulphate) at RT and then once in washing buffer (1X SSC) at RT. The membranes were incubated for 3 h in blocking buffer (Roche) followed by 30 min incubation with Anti-DIG-alkaline phosphatase fAb (Roche) in blocking buffer. The membranes were then washed in DIG washing buffer at RT for 15 min each and then incubated for 5 min in development buffer. CSPD substrate (1∶100 diluted in development buffer) was applied on to the membranes and incubated in dark for 10 min. Chemiluminescence signal was then measured in a Fluorchem machine (Protein Simple) to detect miRNA on the membrane.

### Statistical Analysis

Statistical tests for identifying significant differentially expressed miRNAs were performed using edgeR module with few modifications in the script. The p value cutoff was performed on the data with the significance threshold selected as 0.05.

### miRNA target prediction and pathway analysis

Target prediction and pathway analysis were carried out using protocol reported elsewhere [26]. Briefly, to understand miRNA function in mosquito, we predicted their messenger RNA (mRNA) targets using RNA hybrid tool [33]. 3′UTR of *An. gambiae* downloaded from vectorbase were used for the prediction. The targets showing complementarity with miRNA seed region and binding energy < = −20 Kcal/mol were selected. KOBAS analysis was performed to identify significant pathways targeted by individual miRNAs keeping the threshold pvalue≤0.05 [34]. These selected targets were further used to generate miRNA: mRNA interaction network, visualized by Cytoscape [35].

## Results

Small RNA sequencing using Illumina Truseq chemistry was performed on *An. stephensi* mosquito samples under five different conditions. Small RNA libraries enriched for miRNAs were constructed following manufacturer's instructions from sugar fed naive female mosquitoes 5–6 days old (SF), female mosquitoes at 42 hours (BF 42 h) and 5 days post blood feeding (BF 5d) and from female mosquitoes at 42 hours (iBF 42 h) and 5 days post infected blood feeding (iBF 5d). Post library quantification and cluster generation, the libraries were sequenced using sequencing by synthesis technology, (Illumina Inc.) and the raw reads analysed further using an in-house pipeline developed in our laboratory [26].

### Complexity of small non coding RNAs between libraries as revealed by deep sequencing

**Figure 1:**
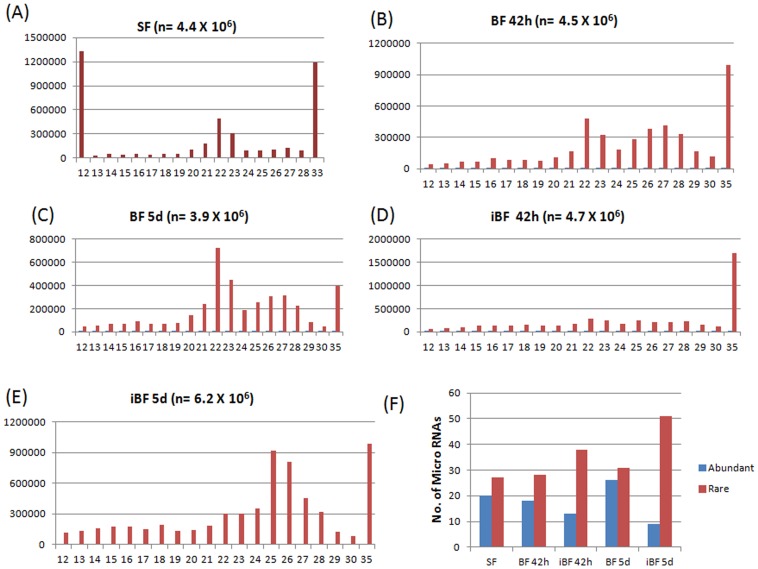
Length distribution of small RNA reads in (A) sugar fed naive female mosquitoes 6–8 days old (SF). (**B**) Female mosquitoes at 42 h post blood feeding (BF 42 h). (**C**) 5 days post blood feeding (BF 5d). (**D**) Female mosquitoes at 42 h (iBF 42 h) and (**E**) 5 days post infected blood feeding (iBF 5d). X axis represents small RNA read lengths in base pairs while Y axis represents number of reads. (**F**) Distribution of abundant and rare miRNAs in all samples. Blue and Red bars represents abundant and rare miRNAs respectively.

Sequencing of all five libraries yielded 2.5×10^7^ reads. After trimming the adaptors, 2.38×10^7^ (94%) of total reads were utilized for further processing (Table 1). A total of 4.4×10^6^, 4.5×10^6^, 3.9×10^6^, 4.7×10^6^, 6.2×10^6^ sequences from SF, BF 42 h, BF 5d, iBF 42 h and iBF 5d libraries respectively were utilized for *in silico* analysis (Figure 1). Among the libraries size distribution of reads varied between 12–35 nt. We found bimodal distribution with peaks at 20–23 nt and again at 32–35 nt in all libraries. 20–23 nt peak corresponds to miRNAs, while 32–35 nt peak represents longer piRNA-like small RNAs. 32–35 nt small RNAs are twice as abundant in mosquitoes at 5 days post infected blood feeding suggesting a possible role of longer sized small RNAs in mosquito during parasite maturation. Whereas, differently sized small RNAs are equally distributed in blood fed and infected blood fed mosquitoes at 42 h post feeding (Figure 1).

**Table 1:** Composition of small RNA in sugar fed naive female mosquitoes 6–8 days old (SF), blood fed female mosquitoes at 42 hours (BF 42 h), 5 days post blood feeding (BF 5d), female mosquitoes at 42 hours (iBF 42 h) and 5 days post infected blood feeding (iBF 5d).

Sample Name	Raw reads	Reads post adaptor trimming	18–30 nt reads	ncRNA reads	Reads mapped to known miRNA (%)	Unique small RNA reads
SF	5478556	4426195	2883395	199021	786128 (21)	244917
BF 42 h	4635098	4554220	4132388	410203	769570 (18)	954582
iBF 42 h	4862848	4795865	4150299	817275	257887 (6)	1267813
BF 5d	4000410	3908552	3489024	223131	1020270 (29)	558168
iBF 5d	6434830	6207543	5290072	651297	283816 (5)	1742904
TOTAL	25411742	23892374	19945178	2300927	3117671	4768384

Small RNA reads between 18–30 nt, accounting for 78% of total reads were selected for further analysis. After removing redundant sequences, a total of 2.4×10^5^, 9.5×10^5^, 12×10^5^, 5.5×10^5^, 17×10^5^ unique sequences were found in SF, BF 42 h, iBF 42 h, BF 5d and iBF 5d libraries respectively. Total of 2.3×10^6^ reads gave hit with database containing ncRNAs (rRNA, tRNA, piRNA, snoRNA) from all organisms. Remaining sequences were then analysed for known and novel miRNA candidates (Table 1).

### Identification of known and novel miRNAs in *An. stephensi* by Deep sequencing

We identified a total of 109 known miRNAs in all five libraries with 100% match with known miRNAs from eight insect species of which 103 miRNAs were conserved in mosquitoes (Table 2). A relative comparative distribution of these 103 mosquito conserved miRNAs is as follows: 65, *An. gambiae*; 87, *Ae. aegypti* and 67, *Culex*. Twelve miRNAs were identified in all eight insect species used in our analysis, illustrating evolutionary conservation of miRNAs across Arthropods (Table 2). Six miRNAs (miR-2796-5p, miR-2796-3p, miR-2779, miR-133-5p, miR-190-3p, miR-iab-8) identified in our study had homologues with mature miRNAs from insect species other than mosquitoes (Table 2).

**Table 2:** List of known miRNAs identified in *An. stephensi*.

S. No	Length	Sequence	miRNA	Tags Per Million (TPM)					aga	aae	cqu	dme	bmo	api	ame	tca
				SF	BF 42 h	iBF 42 h	BF 5d	iBF 5d								
1	23	CCGGTTTTCATTTTCGATCTGAC	as-bantam-5p	43.4	35.1	15.6	75.9	10.2		Y	Y					
2	21	TGAGGTAGTTGGTTGTATAGT	as-let-7	1301	543.2	470.5	1579.3	187.8	Y	Y	Y			Y		
3	22	TGGAATGTAAAGAAGTATGGAG	as-miR-1-3p *	196.4	121.2	76.2	400.2	17.7	Y	Y	Y	Y	Y	Y	Y	Y
4	22	ACCCTGTAGATCCGAATTTGTT	as-miR-10-5p *	9310.6	3068.3	1193.7	6265.6	708.9	Y	Y	Y	Y	Y	Y	Y	Y
5	22	CATCACAGTCTGAGTTCTTGCT	as-miR-11	1269.3	2464.6	807.9	2179	535.9	Y	Y	Y	Y				
6	21	TGAGATTCTACTTCTCCGACT	as-miR-1175-3p	66.8	62.1	11.1	271.9	77.5	Y	Y	Y					
7	22	AAGTGGAGTAGTGGTCTCATCG	as-miR-1175-5p	4.1	6	0.6	11.2	3.8		Y	Y					
8	22	TCCCTGAGACCCTAACTTGTGA	as-miR-125-5p	193.4	121	63.3	200.9	26.2		Y						
9	23	TGAGTATTACATCAGGTACTGGT	as-miR-12	438.8	363.3	110.2	1001.3	151.2	Y	Y	Y	Y			Y	Y
10	22	TTGGTCCCCTTCAACCAGCTGT	as-miR-133-3p	129.4	65.8	63.5	205.2	12.5	Y	Y	Y	Y	Y		Y	Y
11	23	TATCACAGCCATTTTGACGAGTT	as-miR-13-3p	124.8	87.3	51.8	233.7	42.2	Y	Y	Y	Y				Y
12	22	TCGTAAAAATGGTTGTGCTGTG	as-miR-13-5p	18.6	11.8	3.9	21.7	3.5		Y						
13	22	TATTGCTTGAGAATACACGTAG	as-miR-137	39.6	6.6	8.6	21.4	0.6	Y	Y	Y	Y		Y		
14	22	TCAGTCTTTTTCTCTCTCCTAT	as-miR-14 *	4857.6	2869.1	1117.4	3950.3	500.7	Y	Y	Y	Y	Y	Y	Y	Y
15	20	TGAAATCTTTGATTAGGTCT	as-miR-1890	38.6	24.1	13.1	51.7	4.8	Y	Y	Y					
16	23	CTTGGCACTGGGAGAATTCACAG	as-miR-263b-5p	137.4	37.9	16.4	142.2	3.8	Y	Y		Y	Y			
17	21	TCGGTGGGACTTTCGTCCGTT	as-miR-278	24.2	15.3	11.9	38.9	3.4	Y	Y	Y	Y			Y	
18	20	TGACTAGATCCACACTCATT	as-miR-279	176.1	351.2	112.6	418.9	101.1	Y	Y	Y	Y	Y		Y	Y
19	22	TAGCACCATTCGAAATCAGTAC	as-miR-285	613.1	106.5	117.2	396.4	3.2		Y	Y					
20	22	GAAGGAACTTCTGCTGTGATCT	as-miR-2944a-5p	0.3	275.5	98.7	19.7	3.1		Y						
21	19	TGACTAGAGGCAGACTCGT	as-miR-2945	4.7	4	2.6	10.4	0.4		Y						
22	21	TATCACAGCCAGCTTTGAAGA	as-miR-2a	601.6	402.3	189.1	900.4	118.1		Y	Y					
23	24	TATCACAGCCAGCTTTGATGAGCT	as-miR-2b	225.7	115.4	36.6	239.7	36.5		Y						
24	22	CGGCACATGTTGGAGTACACTT	as-miR-305-3p	21.9	55.2	12.9	81.9	7.9		Y	Y					
25	21	ATTGTACTTCATCAGGTGCTC	as-miR-305-5p	277.6	280.4	103.6	522.9	110.6	Y	Y	Y	Y	Y		Y	Y
26	21	TGGCAAGATGTTGGCATAGCT	as-miR-31	63.7	22.2	16.4	97.4	11.9		Y	Y					
27	22	TTTGTTCGTTTGGCTCGAGTTA	as-miR-375	145.1	51.1	58.8	116.2	17.5	Y	Y	Y					
28	22	TCTCACTACCTTGTCTTTCATG	as-miR-71-3p	98	148.6	63.5	372.7	24.2		Y	Y					Y
29	22	AGAAAGACATGGGTAGTGAGAT	as-miR-71-5p	8.3	7.3	4.7	8.7	1		Y	Y					
30	23	GCTTTGGCGCTTTAGCTGTATGA	as-miR-79-5p	0.5	0.2	0.4	0.2	0.1		Y	Y	Y				
31	23	TAATACTGTCAGGTAAAGATGTC	as-miR-8-3p	24812.3	23665.7	7532.4	38198.8	6998	Y	Y	Y	Y	Y		Y	Y
32	22	CATCTTACCGGGCAGCATTAGA	as-miR-8-5p	2144.5	2651.5	817	2713.4	745.9		Y	Y	Y	Y			Y
33	22	AATTGCACTTGTCCCGGCCTGC	as-miR-92b	218.3	677.2	171.9	521.1	89.6	Y	Y	Y					Y
34	22	TGAAACCGTCCAAAACTGAGGC	as-miR-957	819.5	358.3	331.2	1872.3	51.1	Y	Y	Y	Y				
35	22	TAAGCGTATAGCTTTTCCCATT	as-miR-965	2	0.6	1	0.9	0.4	Y	Y	Y					
36	21	TCATAAGACACACGCGGCTAT	as-miR-970	451.9	430.4	150.1	1071.3	117.6	Y	Y	Y	Y				Y
37	19	TAGCTGCCTAGTGAAGGGC	as-miR-980	37.7	17.2	6.9	29.7	7.4		Y	Y					
38	22	TTCGTTGTCGACGAAACCTGCA	as-miR-981	49.1	21.3	19.3	124.4	1.2	Y	Y	Y	Y				Y
39	22	CCCCTTGTTGCAAACCTCACGC	as-miR-988-3p	27.5	10.5	6.3	6.7	2.3	Y	Y	Y	Y				
40	21	TGTGATGTGACGTAGTGGTAC	as-miR-989	55.1	886.4	255.8	519.6	142.3	Y	Y	Y					Y
41	22	TGTTAACTGTAAGACTGTGTCT	as-miR-999	3492.3	1525.9	1052.6	7338.7	412.9		Y	Y	Y				
42	23	TCTTTGGTTATCTAGCTGTATGA	as-miR-9a	1049.7	913.2	340.5	1525.5	307	Y	Y		Y	Y	Y	Y	Y
43	22	TAAAGCTTTAGTACCAGAGGTC	as-miR-9c-3p	11.1	17.9	11.1	9.9	4.5		Y	Y					
44	22	TCTTTGGTATTCTAGCTGTAGA	as-miR-9c-5p	1565.7	3550.9	1051.6	5142.9	1084.7	Y	Y	Y	Y				
45	22	ACGTATACTGAATGTATCCTGA	as-miR-iab-4	2.1	4.9	1	4.4	1.5	Y	Y	Y	Y	Y		Y	Y
46	22	TGAGATCACTTTGAAAGCTGAT	as-bantam-3p	25521.6	14463.9	5316	32017.4	4641.1	Y							
47	22	TCAGATCTACTTCATACCCATG	as-miR-1174	20.6	16.1	2.8	80.2	16.7	Y							
48	20	TAAGGCACGCGGTGAATGCC	as-miR-124 *	45	6.4	13.9	59.7	1.7	Y	Y	Y	Y	Y	Y	Y	Y
49	20	CACATTACAGATTGGGATTA	as-miR-1889	0.9	0.6	0.6	4.7	1.2	Y							
50	22	TGAGGAGTTAATTTGCGTGTTT	as-miR-1891	85.4	54.1	38.2	94.9	2.6	Y	Y	Y					
51	22	TCAGGTACCTGAAGTAGCGCGC	as-miR-275 *	711.3	1860.3	520.4	1011.8	234.5	Y	Y	Y	Y	Y	Y	Y	Y
52	22	TAGGAACTTCATACCGTGCTCT	as-miR-276-3p *	4702.5	5552.2	2553.8	8242.4	1149.8	Y	Y	Y	Y	Y	Y	Y	Y
53	22	TAAATGCACTATCTGGTACGAC	as-miR-277 *	5776.7	2763	2080.6	6081.1	909.7	Y	Y	Y	Y	Y	Y	Y	Y
54	22	TGTCATGGAATTGCTCTCTTTA	as-miR-281-3p	29	121	28.9	263.2	92.7	Y	Y	Y					
55	19	AAATATCAGCTGGTAATTC	as-miR-283	5.4	8.6	2.8	20.9	2.9	Y				Y			
56	22	TCAGGTACTGGATGACTCTCAG	as-miR-306	2160	6349.1	1629.4	5268.4	1285.3	Y							
57	20	TCACAACCTCCTTGAGTGAG	as-miR-307a-3p	1	0.8	0.2	1.9	0.3	Y			Y	Y			Y
58	18	AATCACAGGAGTATACTG	as-miR-308	24.4	21.5	6.9	70.4	10.8	Y	Y	Y					
59	25	TGAACACATCTGGTGGTATCTCAGT	as-miR-317	1900.8	1519.9	547.2	1555.8	343.2	Y							
60	19	GTGAGCAAATATTCAGGTG	as-miR-87	1.2	1.2	0.4	3.2	0.7	Y							
61	20	TATTGCACTTGTCCCGGCCT	as-miR-92a	91.2	51.3	16.2	118.4	18.6	Y	Y					Y	
62	24	GAAGCTCGTTTCTATAGAGGTATC	as-miR-993-3p	28.4	10.1	6.5	38.9	4	Y		Y					
63	20	TGACTAGATTACATGCTCGT	as-miR-996	315.7	396.7	133.2	906.9	146.3	Y	Y	Y					
64	24	AGATATGTTTGATATTCTTGGTTG	as-miR-190-5p *	56.7	51.5	31.6	129.9	9.4	Y	Y	Y	Y	Y	Y	Y	Y
65	20	GTAGGCCGGCGGAAACTACT	as-miR-2796-3p •	25.7	17.2	11.5	79.9	2.9					Y	Y	Y	Y
66	22	TTTAGAATTCCTACGCTTTACC	as-miR-927-5p	934	169.3	133.2	582.4	32.7	Y			Y	Y	Y	Y	Y
67	21	ATATTGTCCTGTCACAGCAGT	as-miR-1000	54	17.9	22.4	111.9	6	Y	Y		Y		Y		Y
68	23	TTTTGATTGTTGCTCAGAAAGCC	as-miR-315-5p	980.5	207.1	231.7	1168.3	22.6	Y	Y	Y	Y		Y	Y	Y
69	23	CAAATTCGGTTCTAGAGAGGTTT	as-miR-10-3p	102	129	44	184.2	24.8			Y	Y	Y			Y
70	22	TGGACGGAGAACTGATAAGGGC	as-miR-184 *	16430.4	13444.8	4872.2	26696	3593.4	Y	Y	Y	Y	Y	Y	Y	Y
71	18	TACTTCTTTACATTCCAT	as-miR-1-5p *	1.4	0.4	0.2	2.4	0.4	Y	Y	Y	Y	Y	Y	Y	Y
72	22	CTAAGTACTAGTGCCGCAGGAG	as-miR-252-5p	1109.7	574.7	311.9	1019.3	163.9			Y	Y	Y			Y
73	18	ATCCGGCTCGAAGGACCA	as-miR-2779 •	0.7	1.2	0.4	0.2	0.1					Y			
74	22	AAGAGAGCTATCCGTCGACAGT	as-miR-281-5p	13394.7	32243.7	6493.9	73962.6	14442		Y	Y	Y	Y			Y
75	23	ATAAAGCTAGATTACCAAAGCAT	as-miR-79-3p	3.8	3	0.8	4.4	0.9	Y	Y		Y	Y		Y	Y
76	21	ACAAGTTTTGATCTCCGGTAT	as-miR-125-3p	10.9	11.6	6.7	16.4	3.7			Y	Y				
77	21	CTTGTGCGTGTGACAACGGCT	as-miR-210-3p	291.1	133.3	131.1	564.4	9.7	Y	Y	Y					
78	21	CTGCTGCCCAAGTGCTTATCG	as-miR-252-3p	4.3	1.9	1.6	3.9	0.7		Y	Y					
79	23	TGGCAGTGTGGTTAGCTGGTTGT	as-miR-34	657.2	848	514.3	1578.3	373.9	Y	Y		Y	Y			Y
80	22	CAAAGCGTTTGGATTCTGAAAC	as-miR-927-3p	126.6	66.6	47.9	167.9	10.1	Y	Y		Y	Y	Y	Y	Y
81	21	AAATTGACTCTAGTAGGGAGT	as-miR-929-5p	15.5	6.4	5.9	18.4	1		Y		Y	Y	Y	Y	Y
82	22	TACCCTGTAGTTCCGGGCTTTT	as-miR-993-5p	15.6	6	4.7	18.7	2.7		Y	Y	Y				
83	21	TAGCACCATGAGATTCAGCTC	as-miR-998	38.3	29.9	10.6	33.7	8.2		Y	Y	Y				
84	22	AACCCGTAGATCCGAACTTGTG	as-miR-100	7992.6	1746.4	947.1	2613.9	464.6	Y	Y	Y		Y		Y	Y
85	22	AATGGCACTGGAAGAATTCACG	as-miR-263a-5p	4314.2	36379.1	9481.8	10687.9	3279.1		Y	Y					Y
86	22	AGCGAGGTATAGAGTTCCTACG	as-miR-276-5p	29.2	28.4	11.7	60.4	8.5	Y	Y	Y		Y			Y
87	21	GTGCATTGTAGTTGCATTGCA	as-miR-33	0.5	0.8	0.4	3.4	0.4		Y	Y		Y		Y	Y
88	21	TGGAAGACTAGTGATTTTGTT	as-miR-7 *	2.9	17	11.3	64.4	1.3	Y	Y	Y	Y	Y	Y	Y	Y
89	23	TCAATTCCGTAGTGCATTGCAGT	as-miR-932	80.8	18.1	24.6	95.4	2.6		Y	Y	Y	Y		Y	Y
90	20	TTACGTATACTGAAGGTATA	as-miR-iab-8 •	0.1	0.8	0.2	0.4	0.1				Y	Y			Y
91	20	TACTGGCCTACTAAGTCCCA	as-miR-193	1	0.2	0.4	0	0		Y		Y				
92	20	TGATTGTCCAAACGCAATTC	as-miR-219	0.3	0	0	1.7	0	Y	Y		Y		Y		Y
93	22	TGGTAACTCCACCACCGTTGGC	as-miR-2765	0.5	0	0	0.9	0		Y			Y		Y	Y
94	25	TGACTAGACCGAACACTCGCGTCCT	as-miR-286a	0.1	0	0.2	1.7	0	Y	Y						
95	21	TGACTAGACCGAACACTCGTA	as-miR-286b	0	5.3	1.4	4.9	0.9		Y						
96	22	TATCACAGTAGTTGTACTTTAA	as-miR-2944a-3p	0	8.8	2.2	0.4	0.1		Y						
97	21	TTTCGAGCAGTAATCAAAGTC	as-miR-315-3p	0	0	0	0.2	0		Y						
98	21	GTGTGCTTTGTGACAATGAGA	as-miR-988-5p	0	0.4	0.2	0.4	0		Y						
99	22	TAGCCTCTTCTAGGCTTTGTCT	as-miR-282	0.9	0	0.4	0.2	0.1	Y							
100	22	TCACTGGGCAAAGTTTGTCGCA	as-miR-309	0	60.4	40	2.9	1.7	Y	Y						
101	20	TCCCTAACGGAGTCAGATTG	as-miR-929-3p	0.3	0	0.2	0	0.1	Y			Y				
102	22	TATCACAGCAGTAGTTACCTGA	as-miR-2944b-3p	0	0.2	0.2	0.7	0.3		Y					Y	Y
103	20	CACAACCTCCTTGAGTGAGC	as-miR-307b	0.3	0	0	0.7	0.1		Y				Y		
104	21	AGCTGCTGACCACTGCACAAG	as-miR-210-5p	0.5	0.2	0.2	0.7	0			Y					
105	20	AGCTGGTTGACATCGGGTCA	as-miR-133-5p •	0	0.2	0	0.7	0				Y				Y
106	22	CCCAGGAATCAAACATATTATT	as-miR-190-3p •	23.3	0	0.2	0.7	0.1				Y	Y			Y
107	18	TCACAGCCAGCTTTGATG	as-miR-2c *	0	2.5	1	1.4	0.4	Y	Y	Y	Y	Y	Y	Y	Y
108	22	AGGGGTTTCTTTCGGCCTCCAG	as-miR-2796-5p •	0.1	0	0	0	0				Y	Y			Y
109	20	ACTCACTCAACCTGGGTGTG	as-miR-307a-5p	0.1	0	0.2	0.4	0	Y	Y		Y	Y	Y	Y	Y

aga  =  *Anopheles gambiae*; aae  =  *Aedes aegypti*; cqu  =  *Culex*; dme  =  *Drosophila melanogaster*; bmo  =  *Bombyx mori*; ame  =  *Apis mellifera*; api =  *Acyrthosiphon pisum*; tca  =  *Tribolium castaneum*.

*miRNAs present in all eight insect species.

•miRNAs not previously identified in mosquitoes.

**Figure 2:**
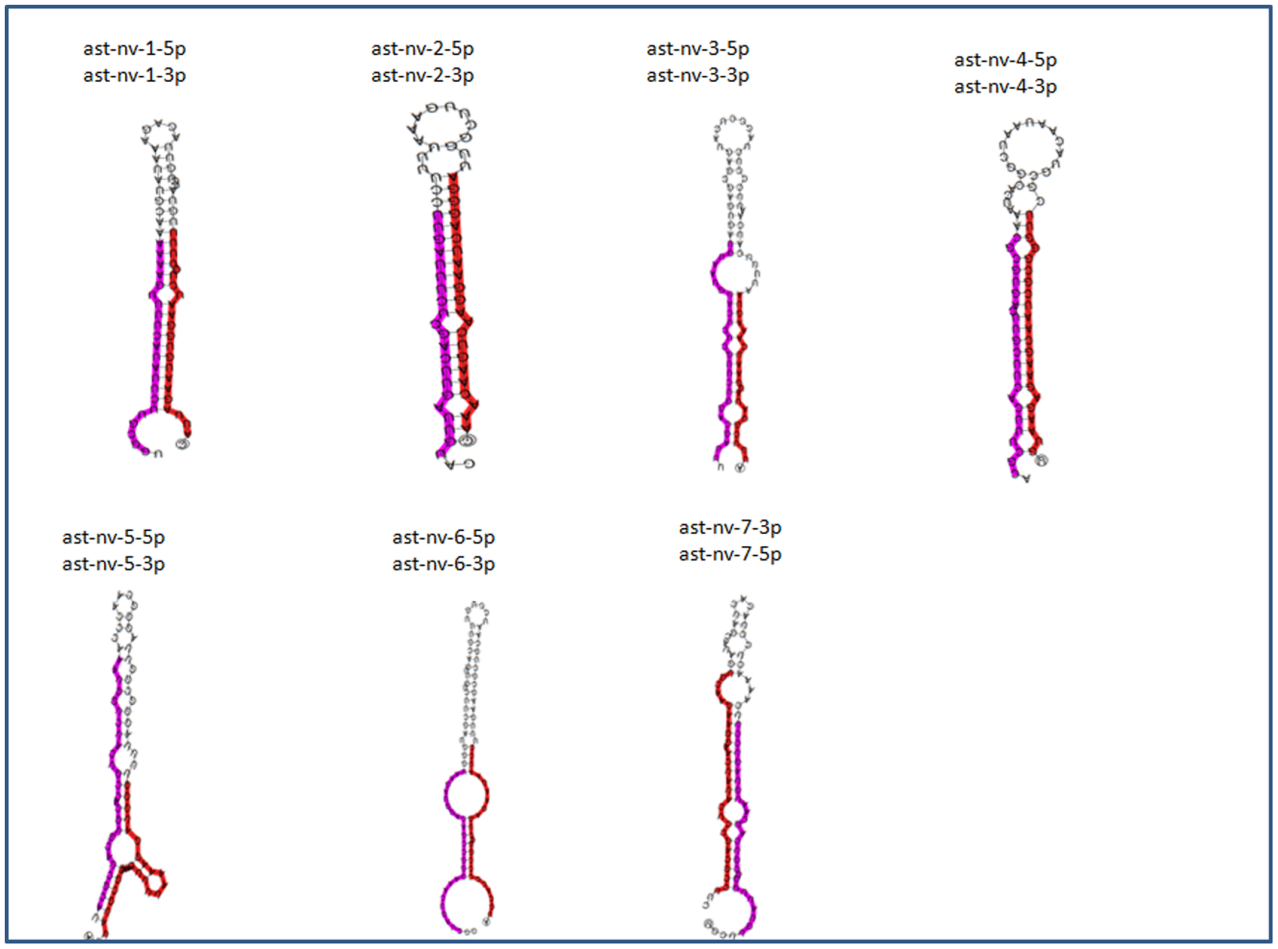
Predicted secondary structures of Novel miRNAs. Precursor sequences were folded using RNA fold. Coloured sequences (Pink = −5p; Red = −3p) represent mature miRNAs on both arms of the precursor miRNA.

**Figure 3:**
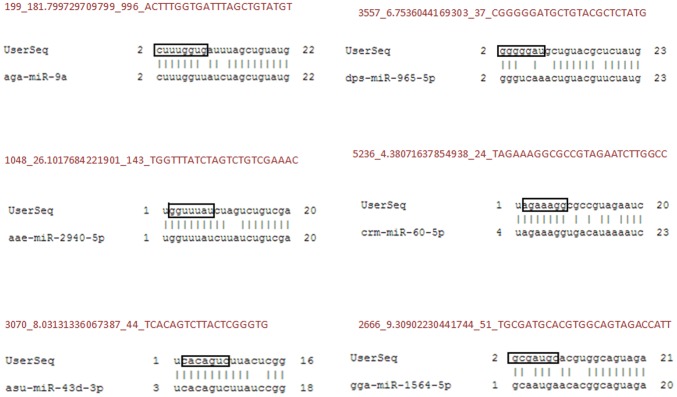
Family-wise clustering of novel miRNAs. Alignment results of novel miRNA sequences with known mature miRNAs present in mirbase. Seed region of Novel miRNAs are enclosed in a box.

High throughput small RNA sequencing data analysis identified several novel miRNAs in *An. stephensi*. Using our pipeline for novel miRNAs identification, 17 small RNA sequences showed all characteristic signatures of a miRNA (Figure 2 and Figure S1) as described earlier [36]. These miRNA sequences have been named in a specific order as detailed in Table 3. miRNAs found on 5′ and 3′ arms of the precursors were annotated as -5p and -3p respectively. These sequences were further tested for hairpin formation and seven sequences showed complementary 5p and 3p miRNAs elevating these sequences as bonafide miRNAs (Table 3). NCBI BLAST was performed with these sequences and the results showed high conservation of these sequences across species. In order to categorize the novel miRNAs into known miRNA families, the sequences were blasted against mirBase, a database containing sequences of known miRNAs. Six novel miRNAs were tentatively classified into known miRNAs families as they showed highly conserved seed region with known miRNAs from other species. Sequence extending beyond seed region also showed high level of conservation with known miRNAs (Figure 3).

**Table 3:** List of novel miRNAs identified in *An. stephensi*.

S. No	Length	Sequence	miRNA	Tags Per Million (TPM)				
		**miRNAs on both arms of Pre-miRNA**		SF	BF 42 h	iBF 42 h	BF 5d	iBF 5d
1	22	ATTAGAATGTGGAATCTGTTTT	as-nv-1-5p	5.2	2.5	2.8	7.9	0.6
2	22	AAAAGTTTTCATATTCTTGCGG	as-nv-1-3p	3.2	1	0.8	1.4	0.3
3	22	AAACAAGTCAAGGAATCAGGGA	as-nv-2-5p	30.8	17.9	6.9	19.2	5.9
4	21	TTGATTTCTCGACTTGATTGT	as-nv-2-3p	16.2	15.5	5.5	28.2	2.1
5	28	TGTAATGTACTCTCGTTCTGGACGATTT	as-nv-3-5p	4.7	643 ¥	200.9	1350.1	177.3
6	21	TTTCGGATATGAATCAAAGTA	as-nv-3-3p	5.2	78.7	35.7	34.4	12.1
7	22	GTAAGAGAAGCAATCGCGGGTT	as-nv-4-5p	520 ¥	356.6	133	817.1	93.3
8	23	CGCGTGACTTGCTTCACTTTCGC	as-nv-4-3p	1	1	0.6	2.7	0.6
9	26	AATGGTCTACTGCCACGTGCATCGCA	as-nv-5-5p	1.8	11.6	6.7	19.7	2.1
10	26	TATGCGGAAACTTTTAAAAGGATGGG	as-nv-5-3p	9.3	9.4	2	8.4	1
11	25	TGCACCCCCGGGCTGAGAAGAATCC	as-nv-6-5p	8	17.6	4.9	39.7	4.6
12	26	TAAGATGGACAGCCGGGAAACTGATC	as-nv-6-3p	2.7	24.1	2.4	19.2	5.5
13	28	TGCAAGAAGGAACTATACTCCGACGCCT	as-nv-7-5p	2.7	2.8	1	4.2	0.4
14	27	TGTATTGGTCGTCAGCAATGTAGTCCT	as-nv-7-3p	56	34.7	9.2	34.4	3.5
		**miRNAs on one arm of Pre-miRNA**						
15	23	ACTTTGGTGATTTAGCTGTATGT	as-nv-8	181.7	529.6	155.8	571.6	193
16	23	TGGTTTATCTAGTCTGTCGAAAC	as-nv-9	26.1	14.8	2.4	26.2	4.6
17	25	TGATGTAATGTACTCTCGTTCTGGA	as-nv-10	10	16.1	8	42.4	6.3
18	27	TATGACCAGAGGATAGATGTGACTACT	as-nv-11	8	22.4	120.7	11.4	141.8
19	23	CGGGGGATGCTGTACGCTCTATG	as-nv-12	6.7	16.1	4.3	10.7	2.4
20	25	TACGAGAAGATGGTCGCAAAAGCTC	as-nv-13	6.3	14	6.1	15.9	6.5
21	24	ACGACGTATGAGGATACCCTGAAA	as-nv-14	5.4	85.4	7.1	17.9	6.3
22	25	TCCTTCTGTTGACCTGGCGCTCGAC	as-nv-15	5.1	26.7	5.3	21.7	4.8
23	19	TCACAGTCTTACTCGGGTG	as-nv-16	8	8.1	2.2	13.4	1.2
24	26	TAGAAAGGCGCCGTAGAATCTTGGCC	as-nv-17	4.3	8.1	3.9	12.7	0.9

¥ novel miRNAs validated by Northern hybridization. miRNA ast-nv-4-5p was validated using sample SF; miRNA ast-nv-3-5p was validated using sample BF 42 h.

### Identification of miRNAs with sequence mismatches

In the present study, identification of *An. stephensi* miRNAs was carried out using small RNA reads with 100% identity with known mature miRNAs. In addition, we performed the analysis with up to two mismatches in the mature miRNA sequences. By this method, we identified the following eight miRNAs (ast-miR-2941-3p, ast-miR-263a-3p, ast-miR-312-3p, ast-miR-316-5p, ast-miR-317-5p, ast-miR-956-3p, ast-miR-971-3p, and ast-miR-3843-3p) (Table S1).

A previous study has identified *An. stephensi* miRNAs using *An. gambiae* genome dataset [25]. In the present investigation, we mapped mature miRNAs of *An. stephensi* identified in this study against the genome of *An. stephensi*, to validate their presence in the mosquito. All 109 miRNAs reported here mapped perfectly with the genome. However, keeping in mind the low coverage of *An. stephensi* genome and possible inaccuracy in genome sequence, we reduced the stringency of our mapping and allowed up to two mismatches and conducted the analysis. We identified three miRNAs (ast-miR-2940-3p, ast-miR-2944b-5p, ast-miR-2951-3p) containing up to 2 mismatches with genome of *An. stephensi* (Table S1), although they show perfect match with mature miRNAs in the current miRBase database [27].

### Validation of known and novel miRNAs in *An. stephensi* by Northern blot

**Figure 4:**
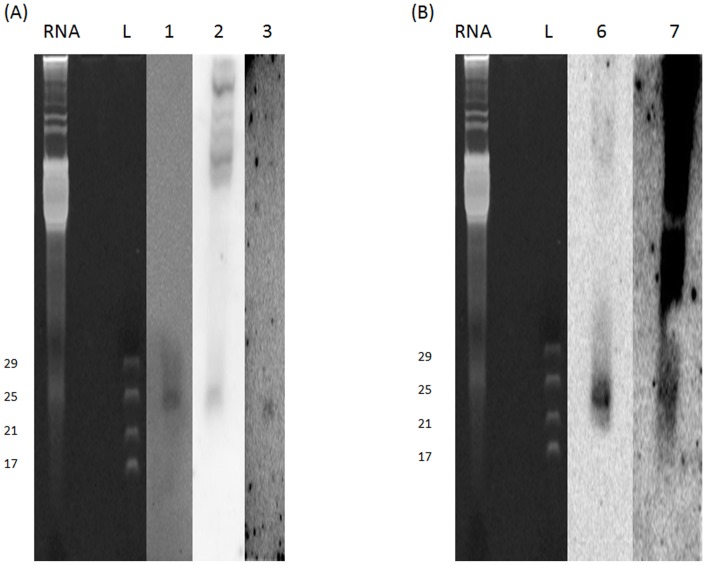
Northern hybridization of *An. stephensi* miRNAs. (**A**) Validated presence of (1) miR-286, (2) miR-309 and (3) miR-989 in *An. stephensi* via DIG based Northern hybridization. (**B**) Northern hybridization of novel miRNAs, (4) ast-nv-3-5p, (5) ast-nv-4-5p in *An. stephensi* with TPM>500. 10 ug of Total RNA from BF 42 h mosquito was used for northern blots of (1), (2), (3) and (4) whereas (5) was validated using total RNA from SF. RNA: Total RNA run on PAGE; L: Ladder.

Having identified miRNAs from the five libraries, efforts were taken to validate some of these miRNAs using Northern hybridisation. Total RNA from BF 42 h was probed using locked nucleic acid (LNA) probes (Exiqon Inc) for miR-286, miR-309, miR-989, selected at random (Figure 4a).

Similarly, candidate sequences showing miRNA characteristics were annotated as novel miRNAs and further validated by Northern hybridisation. From our dataset of predicted miRNAs, five miRNAs were selected in random and Northern hybridisation was performed. Two miRNAs (ast-nv-3-5p, ast-nv-4-5p) possessed TPM (Tags per million) >500 and were detected by Northern hybridization (Figure 4b). The other three miRNAs (ast-nv-2-5p, ast-nv-6-3p ast-nv-7-3p) were not detected probably due to low expression in our experimental samples.

### miRNA abundance in sugar fed, blood fed and infected *An. stephensi*

Distribution and relative abundance of different *Anopheles* miRNAs under three physiological conditions ie, sugar fed, blood fed and parasite infected blood fed was computed. Total number of known miRNAs varied between the libraries of SF, BF and iBF samples. Maximum number of known miRNAs (n = 107) were found in BF 5d while BF 42 h and iBF 5d possessed the least number of miRNAs each (n = 100) (Table 2). Total of 102 and 103 known miRNAs were identified in SF and iBF 42 h respectively (Table 2). Out of 109 known miRNAs, 90 were found in all five libraries (Table 2). miRNAs were classified as “abundant” or “rare” based on the tags per million (TPM) value. TPM >1000 were classified as abundant while those with TPM<10 were classified as rare. In case of SF, 20 miRNAs were classified as abundant while 27 were classified under rare category (Figure 1F). One miRNA, miR-2796-5p was seen exclusively in SF (Table 2). However, the number of reads for this miRNA was very less (<5 reads) and requires further validation. At BF 42 h, 18 and 28 miRNAs were found to be abundant and rare respectively. Three (miR-286b, miR-2944a-3p, miR-309) were expressed significantly in BF 42 h with no reads present in SF, indicating blood meal induced expression of these miRNAs (Table 2). Similarly, 26 were abundant and 31 were rare in BF 5d (Figure 1F). miR-315-3p was exclusively expressed in BF 5d with read count less than five (Table 2).

Furthermore, abundance of individual miRNAs also varied between sugar fed, blood fed and infected female mosquitoes as reflected by the percentage of miRNA reads to the small RNA population in the libraries (Table 1). Our analysis showed significant down-regulation of many miRNAs upon *Plasmodium* infection (iBF 5d). Four miRNAs, namely, bantam-3p, miR-8-3p, miR-184, miR-281-5p were the most abundant in all five libraries (Table 2). miR-263a-5p showed maximum number of reads in BF 42 h and iBF 42 h, miR-bantam-3p was most the abundant miRNA in SF, miR-281-5p in BF 5d and iBF 5d.

We found relatively less number of miRNAs in *Plasmodium* infected mosquitoes, 13 at iBF 42 h and nine in iBF 5d that were abundantly expressed indicating overall down-regulation of miRNA expression post infection in mosquitoes. iBF 5d contained maximum number of miRNAs showing TPM<10 (n = 51) whereas iBF 42 h contained 38 such miRNAs (Figure 1F).

Among six miRNAs (miR-2796-5p, miR-2796-3p, miR-2779, miR-133-5p, miR-190-3p, miR-iab-8) conserved in the eight arthropod species but not conserved in mosquitoes, miR-2796-3p was moderately expressed in all samples with iBF 5d showing maximum expression. miR-190-3p was also moderately expressed in SF with no reads in BF42 h. Remaining four miRNAs showed very less abundance in all the stages (Table 2).

### Temporal regulation of miRNAs post blood feeding and infection in *An. stephensi*

Exploiting the large data and deep coverage from high throughput sequencing of the libraries, we generated miRNAs expression profiles in blood fed and infected female mosquitoes. MiRNAs were profiled at two time points i.e. at 42 hours and 5 days post infected blood feeding coinciding with late phase of midgut invasion by the parasite and initiation of sporozoites release from the oocysts. Using in-house developed Perl script, we calculated TPM for individual miRNAs and compared their expression profiles across different experimental conditions. EdgeR module was used to identify significantly regulated (p value≤0.05) miRNAs between different experimental conditions. The final set of significantly regulated miRNAs was selected on the basis of their p value≤0.05, fold change ≥1.5 and TPM>10.

**Figure 5:**
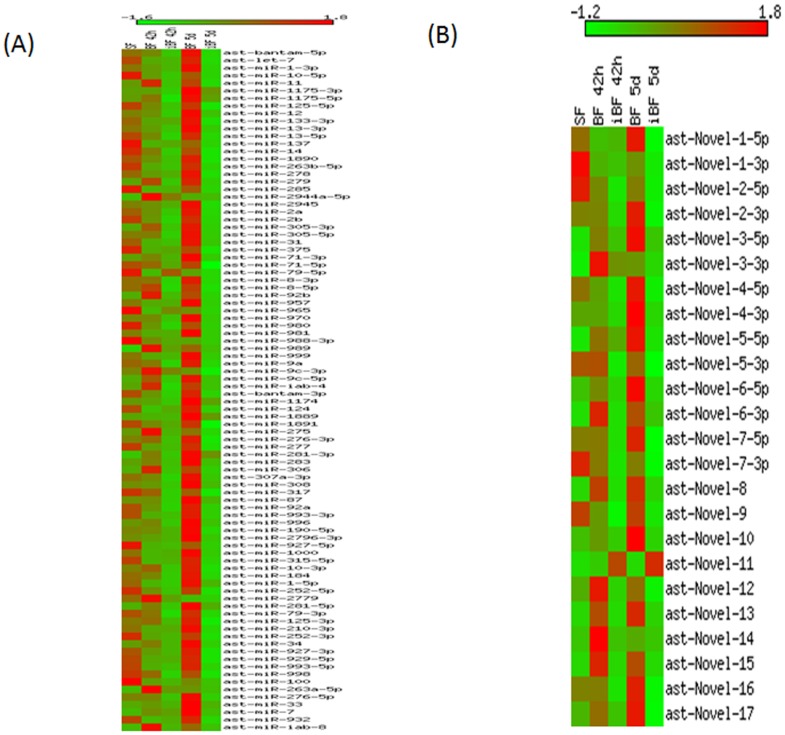
Heat Map of differentially expressed miRNAs present in small RNA libraries of *An. stephensi*. (**A**) Expression profile of miRNAs in sugar fed naive female mosquitoes 6–8 days old (SF), 42 h post blood fed female mosquito (BF 42 h), 42 h post infected blood fed female mosquito (iBF 42 h), 5 days post blood fed female mosquito (BF 5d), 5 days post infected blood fed female mosquito (iBF 5d). Colour gradation from light green to dark red indicates relative increase in miRNA expression. (**B**) Heat Map of differentially expressed novel miRNAs present in all small RNA libraries of *An. stephensi*. Expression profile of miRNAs in sugar fed naive female mosquitoes 6–8 days old (SF), 42 h post blood fed female mosquito (BF 42 h), 42 h post infected blood fed female mosquito (iBF 42 h), 5 days post blood fed female mosquito (BF 5d), 5 days post infected blood fed female mosquito (iBF 5d). Colour gradation from light green to dark red represents relative increase in miRNA expression.

With respect to blood feeding, 11 miRNAs (miR-2944a-5p, miR-92b, miR-989, miR-275, miR-281-3p, miR-281-5p, miR-306, miR-263a-5p, miR-7, miR-309 and miR-305-3p) were significantly up-regulated in BF 42 h compared with SF (Table 2, Figure 5). Whereas, we found only two miRNAs, namely, miR-190-3p and miR-124 down-regulated in BF 42 h compared with SF. In case of BF 5d, most of the miRNAs showed a similar profile with that of SF indicating the similarity in the physiological state of mosquito at this stage (Figure 5). We identified 15 miRNAs that were regulated in BF 5d compared with SF, possibly due to one round of blood digestion and egg production in BF 5d (Table 2).

We noticed distinct variation in expression pattern of miRNAs in the following corresponding conditions, namely 42 hrs and 5 days post *Plasmodium* infection. In order to understand *Plasmodium* stage-specific regulation, the miRNAs were profiled at two specific conditions coinciding with effect of parasite first invasion in *Anopheles* midgut at 42 hrs and late phase of *Plasmodium* development in midgut [37]. It is interesting to note that distinct subsets of miRNAs showed regulation in the sets analysed namely iBF 42 h vs BF 42 h and iBF 5d vs iBF 42 h. In iBF 42 h, four miRNAs (miR-124, miR-137, miR-1000, miR-932) were significantly up-regulated compared to BF 42 h. However, none of the identified miRNAs were down-regulated in iBF 42 h compared to BF 42 h. In iBF 5d, four miRNAs (miR-1175-3p, miR-1174, miR-281-3p and miR-281-5p) were significantly up-regulated, whereas 10 miRNAs (miR-285, miR-2944a-5p, miR-309, miR-210-3p, miR-1891, miR-981, miR-315-5p, miR-932, miR-124 and miR-7) were significantly down-regulated when compared with iBF 42 h showing parasite stage specific expression in mosquito (Table 2, Figure 5).

### Expression profiling of novel miRNAs in blood fed and infected mosquito

TPM values of novel miRNAs were compared between libraries to identify miRNAs that showed regulated expression post blood feeding and infection in mosquito. miRNAs ast-nv-3-3p, ast-nv-3-5p, ast-nv-5-5p, ast-nv-6-3p, ast-nv-8, ast-nv-12 and ast-nv-14 were found to be up-regulated in BF 42 h (Figure 5B, Table 3).

As observed in the case of known miRNAs, most of the novel miRNAs were down-regulated in *Plasmodium* infected mosquito samples. The miRNA ast-nv-11 was up-regulated in iBF 42 h and iBF 5d. Whereas, ast-nv-3-5p, ast-nv-3-3p and ast-nv-5-5p were significantly up-regulated in iBF 42 h compared with SF (Figure 5B, Table 3). ast-nv-6-3p was found to be up-regulated in iBF 5d compared with iBF 42 h. Our analysis also revealed three miRNAs, ast-nv-2-5p, ast-nv-1-3p, ast-nv-7-3p to be significantly down-regulated in all stages of blood fed and infected mosquito compared with SF (Figure 5B, Table 3).

### Validation of miRNA profile by real time PCR

**Figure 6:**
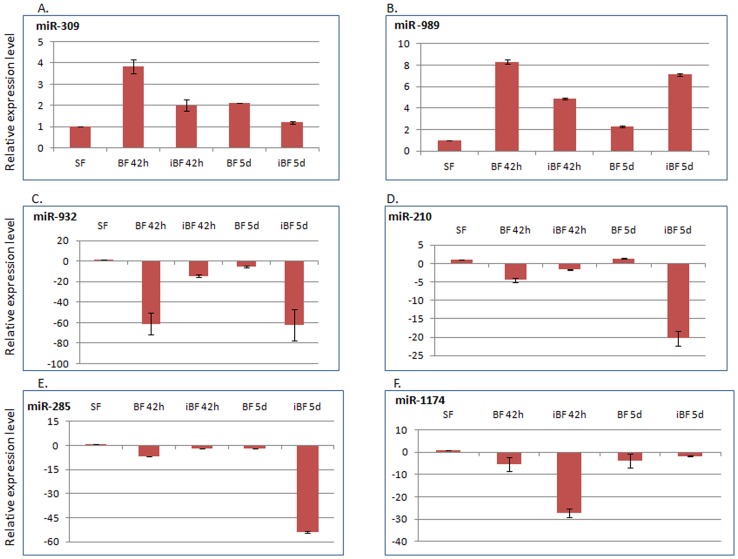
Expression profiling by Real time PCR of up-regulated and down-regulated miRNAs. (**A**) miR-309,(**B**) miR-989,(**C**) miR-932, (**D**) miR-210,(**E**) miR-285 and (**F**) miR-1174 were profiled in sugar fed naive female mosquitoes 6-8 days old (SF), female mosquitoes at 42 hours (BF 42 h) and 5 days post blood feeding (BF 5d) and from female mosquitoes at 42 hours (iBF 42 h) and 5 days post infected blood feeding (iBF 5d). Y axis depicts fold change in miRNA expression in samples compared with sugar fed naive female mosquitoes 6–8 days old (SF), taken as 1.

To verify small RNA sequencing results described above, the expression profile of six differentially expressed miRNAs were validated by quantitative real time polymerase chain reaction (qRT-PCR). All six miRNAs showed similar expression profiles as revealed by small RNA sequencing analysis. Both small RNA sequencing and RT-PCR showed that miR-309 and miR-989 were up-regulated in BF 42 h compared with SF (Figure 6A and 6B). Similarly, miR-309 did not show significant expression difference in iBF 42 h and iBF 5d when compared to SF (Figure 6A) whereas miR-989 was highly up-regulated in both time points of infected mosquito compared to SF (Figure 6B). Both the miR-210 (Figure 6D) and miR-285 (Figure 6E) were exclusively down-regulated at iBF 5d when compared to other experimental conditions. miR-932 was significantly up-regulated at iBF 42 h compared with BF 42 h whereas it was down-regulated at iBF 5d compared to iBF 42 h (Figure 6C). At iBF 5d, miR-1174 was identified to be significantly up-regulated compared to iBF 42 h (Figure 6F). All RT-PCR amplified products were cloned and sequenced to validate amplification of correct miRNAs. BLAST results revealed specific miRNAs amplified by RT-PCR, thereby further validating small RNA sequencing.

### miRNA target prediction and pathway analysis

**Figure 7:**
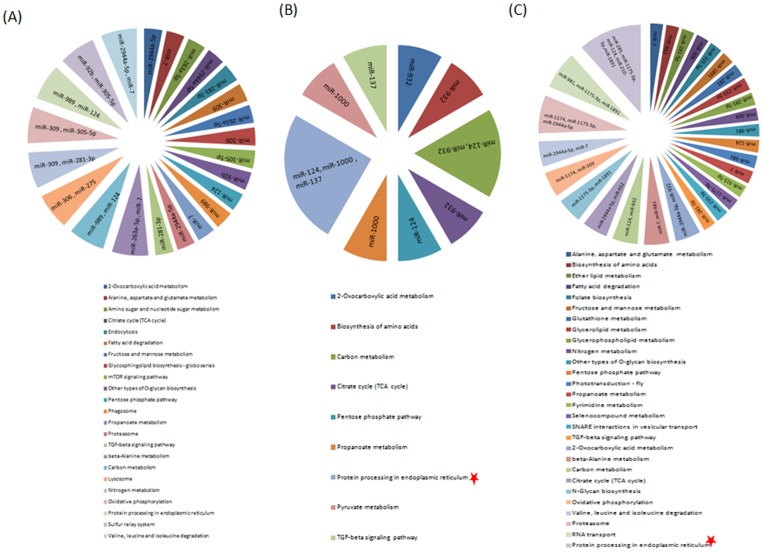
KOBAS analysis of miRNA targets predicted by RNA hybrid. Pie charts represents significant pathways (P value<0.05) targeted by miRNAs regulated at (**A**) BF42 h, (**B**) iBF 42 h and (**C**) iBF 5d respectively. Pie chart area for each pathway represents percentage of regulated miRNAs targeting it. miRNAs are listed in the pie area of respective pathway it is targeting. Pathway marked star are targeted by maximum number of miRNAs.

To understand the function of miRNAs, it is obligatory to study their targets. As miRNAs work in clusters to regulate various biological processes, we aimed to predict targets for all miRNAs that were regulated at different experimental conditions. We predicted targets for all those miRNAs that were significantly modulated, namely, 12 miRNAs in BF 42 h, four miRNAs in iBF 42 h and 14 miRNAs that were regulated in iBF 5d. miR-190-3p was eliminated from target analysis as its reads were not found in BF 42 h though it was moderately expressed in SF and classified as down-regulated miRNAs at BF 42 h. Maximum number of mRNAs were targeted by miR-989 (n = 1222), miR-1000 (n = 741) and miR-281-3p (n = 284) that were up-regulated at BF 42 h, iBF 42 h and iBF 5d respectively (Table S2). miR-124 was the only down-regulated miRNAs at BF 42 h that targeted (n = 505) mRNAs in mosquito (Table S2). Among down-regulated miRNAs at iBF 5d, maximum and minimum number of mRNAs were targeted by miR-124 (n = 505) and miR-7 (n = 99) respectively (Table S2). The targets were analysed by KOBAS and those falling under significant pathways (p value<0.05) were studied. miRNAs regulated at BF 42 h targeted 23 different pathways with eight of them being shared between two different miRNAs (Figure 7A). Two pathways, carbon metabolism and protein processing in endoplasmic reticulum were commonly targeted by down-regulated (miR-124) and up-regulated (miR-989) miRNAs. Post infection, we identified nine and 28 different pathways targeted by miRNAs regulated at iBF 42 h and iBF 5d respectively (Figure 7B and 7C). Among these pathways, two were shared between two or more miRNAs at iBF 42 h and ten different pathways by iBF 5d (Figure 7B and 7C). At iBF 5d, five pathways were commonly targeted by up- and down-regulated miRNAs. Oxidative phosphorylation was targeted by miR-1174 and miR-309, Proteasome was common between miR-1174, miR-1175-3p and miR-2944a-5p, RNA transport commonly targeted by miR-1175-3p, miR-981 and miR-1891, protein processing in endoplasmic reticulum targeted by miR-1175-3p, miR-285, miR-124, miR-210-3p and miR-1891 and N-Glycan biosynthesis was targeted by miR-1175-3p and miR-1891 (Figure 7C). Among all pathways, we identified protein processing in endoplasmic reticulum to be regulated by maximum number of miRNAs namely miR-124, miR-1000 and miR-137 in iBF 42 h and by miR-1175-3p, miR-285, miR-124, miR-210-3p and miR-1891 in iBF 5d (Figure 7B and 7C).

**Figure 8:**
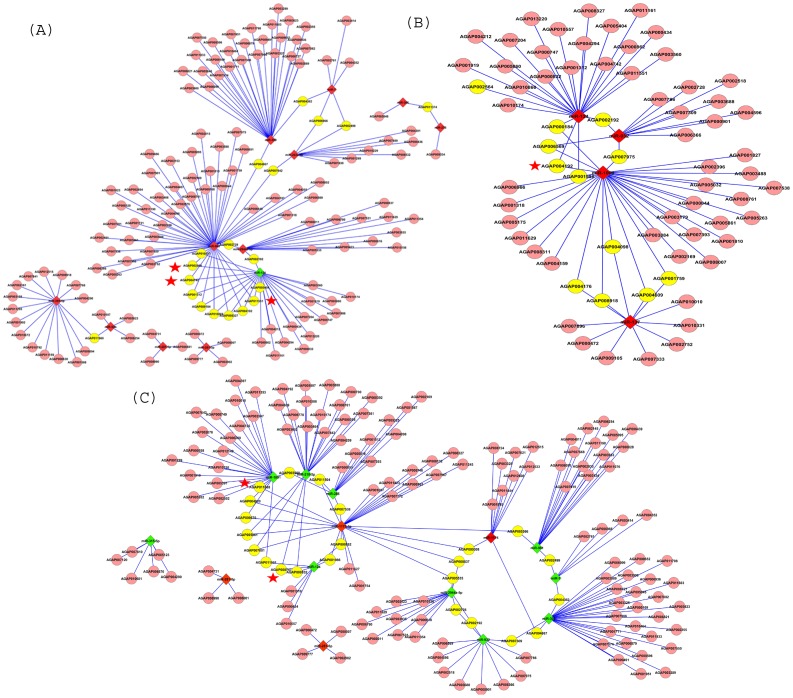
miRNA-mRNA interaction network. Interaction network of regulated miRNAs and their targets at (**A**) BF 42 h, (**B**) iBF 42 h and (**C**) iBF 5d. miRNAs are diamond shaped whereas targets are circular. Up-regulated miRNAs are highlighted in red whereas down-regulated are highlighted in green colour. Transcripts targeted by two or more miRNAs are marked in yellow colour. Significance of the Transcripts marked (*) are explained in the discussion.

miRNA:mRNA interaction was analyzed through network generation as shown in Figure 8. Several transcripts were found to be targeted by two or more miRNAs. At BF 42 h eight transcripts (AGAP002499, AGAP006966, AGAP007642, AGAP011960, AGAP02728, AGAP004867, AGAP004362 and AGAP011374) were targeted by two or more miRNAs up-regulated at BF 42 h (Figure 8A). Whereas, 11 transcripts (AGAP010866, AGAP000184, AGAP001312, AGAP002192, AGAP004742, AGAP011551, AGAP002564, AGAP010557, AGAP005404, AGAP004192 and AGAP008327) were found to be commonly targeted by up- and down-regulated miRNAs at BF 42 h (Figure 8A). At iBF 42 h, two or more up-regulated miRNAs targeted 11 different mRNAs (AGAP000184, AGAP001986, AGAP002192, AGAP004192, AGAP007975, AGAP006569, AGAP008918, AGAP004098, AGAP004609, AGAP001759 and AGAP004176) (Figure 8B). Target analysis at iBF 5d revealed 13 mRNAs (AGAP007538, AGAP011504, AGAP005535, AGAP008837, AGAP004867, AGAP000862, AGAP004020, AGAP001986, AGAP007001, AGAP005861, AGAP005366, AGAP011888 and AGAP009570) common between up- and down-regulated miRNAs (Figure 8C). Furthermore, we identified nine mRNAs (AGAP002499, AGAP000832, AGAP000747, AGAP003560, AGAP002728, AGAP002192, AGAP011551, AGAP004362 and AGAP007309) and one mRNA (AGAP000308) were targeted by up- and down-regulated miRNAs at iBF 5d respectively (Figure 8C).

## Discussion

RNA interference is the most important and primary defence employed by insects to protect themselves from pathogens. Several components of this important phenomenon are involved in various biological processes, miRNAs being the most important of such molecules. Several miRNAs have been identified in various mosquito species and some of their roles in insect reproduction and infection have been explained [6], [25]. However, their role in *Plasmodium* development is poorly understood. This study was conducted to identify and elucidate role of miRNAs post blood feeding and *Plasmodium* infection in *An.stephensi*.

The present study is the first report where small RNA reads were mapped on to *An. stephensi* contigs for identification of novel miRNAs which has led to the identification to a large number of known and novel miRNAs. Seven pairs (-5p and -3p) of miRNAs were identified that fulfilled all criteria required to be classified as a miRNA [36]. Few were further classified into known miRNA families based on seed homology. Additionally, some of novel miRNAs identified lacked any sequence homology with known miRNAs, thereby identifying new families of miRNAs that might play important role during *Plasmodium* development in vector.

To elucidate the role of miRNAs listed in this study, information related to their expression profile post blood feeding and infection is necessary. Here, expression profile of miRNAs at two time points post blood feeding and parasite infection were investigated using small RNA sequencing and further validated by qRT-PCR. In our study, we found significant down-regulation of miRNAs post infection of mosquito by *Plasmodium* parasite. Due to co-evolution of host-parasite crosstalk, hosts are known to resist parasite infection whereas parasite manipulate host to increase its chances of survival and transmission rates [38]. Also, *Plasmodium* infection is known to alter transcriptome and proteome profile in mosquito [4], [39]. Therefore, miRNA regulation in our study could have been brought about by targeting the components involved in host miRNA biogenesis pathway either by molecules present in blood or the invading parasite. Keeping in view the process of establishment of parasite in insect, we hypothesize parasite derived factors might play a role in the regulation of miRNAs involved in defense response of host against invasion.

Blood feeding in hematophagus insects, such as mosquito is essential for reproduction and is exploited by *Plasmodium* parasite to complete its life cycle. Identification of miRNAs playing role in insect reproduction is crucial to control mosquito population and hence parasite transmission from one host to another. We identified few miRNAs that were up-regulated in mosquito post blood feeding. Such induced expression indicates towards their involvement in mosquito reproduction. In the present investigation miR-275 was up-regulated upon blood meal ingestion. Employing anti-miRNA in feeding experiments, miR-275 was shown to play a direct role in blood digestion and egg development in *Ae. aegypti* [40]. The observed up-regulation of miR-275 in present set of experiments also suggests its role in blood digestion and egg development and correlates with the observed physiological situation. Another study on *Aedes* reported induced expression of aae-miR-375 in presence of blood meal [41]. We did not identify miR-375 regulated at BF 42 h when compared with SF, probably due to insect specific regulation of this miRNA. In addition to these miRNAs, miR-309 was found to be induced by blood feeding in mosquito. This miRNA has been implicated in facilitating normal embryo development in *Drosophila* and may be involved in similar biological process in *An. stephensi* [22], [23]. Also, miR-309 was not identified in SF in our analysis that correlates with previous study where it was identified to be involved in turnover of maternal mRNA during maternal to zygotic transition in fly [42]. Another miRNA, miR-989 was shown to be up-regulated post blood feeding in mosquitoes and was abundantly expressed in ovaries of blood fed mosquitoes [6], [25]. Our analysis also revealed up-regulation of this miRNA in small RNA sequencing and RT-PCR analysis of BF 42 h mosquito.

In this study, miRNAs were profiled at two time points i.e. at 42 hours and 5 days post infected blood feeding coinciding with late phase of midgut invasion by the parasite and initiation of sporozoites release from the oocysts. Previous study [6] has shown that miR-1174 and miR-1175 were down-regulated in midgut tissue at 24–48 hrs post infected blood feeding. Our study showed up-regulation of the above mentioned miRNAs in whole body of mosquitoes at iBF 5d. The same study also identified miR-989 up-regulated in midgut of infected female mosquito. However, in our study, miR-989 was not significantly regulated at iBF 42 h whole body compared with BF 42 h. The reason for such discrepancy could be the different tissues, namely midgut used for profiling of these miRNAs in above mentioned study whereas our study was performed on whole body tissues. Additionally, two miRNAs (miR-281-5p and miR-281-3p) were up-regulated at iBF 5d compared with iBF 42 h. These miRNAs may have parasite stage specific function through Toll pathway as miR-281 has putative targets involved in Toll pathway in *D. melanogaster* [43]. Same study identified multiple genes involved in immune pathways of *D. melanogaster* as putative targets for miR-281, miR-124, miR-315, miR-309 and miR-210 that were found to be regulated in mosquitoes during parasite maturation in this study.

Numbers of genes are altered at transcriptome as well as proteome level during the course of blood feeding and parasite development in the mosquito [4], [39]. Few of these altered molecules might serve as agonist or antagonist against *Plasmodium* development. A number of factors are known to regulate expression of such genes including miRNAs. Therefore, in order to link miRNA regulation observed in this study with mRNAs that might play role during egg development and parasite life cycle, we carried out preliminary target prediction of selected miRNAs. Each miRNA targeted multiple transcripts. Many of these transcripts were targeted by two or more miRNAs. In mosquito, blood meal is required for initiation of egg maturation involving numerous pathways. One such pathway is TOR signalling pathway, known to activate production of yolk protein precursors for egg development in blood fed mosquito [44]. This pathway was found to be targeted by miR-305-5p, highlighting its importance in events occurring post blood feeding in mosquito. Blood feeding in mosquito also induces major metabolic challenge including change in redox homeostasis due to rapid blood digestion and assimilation of nutrients present in the blood meal. In our study, miRNAs regulated post blood meal were identified to target mRNAs involved in amino acid metabolic pathways required for oogenesis and preventing accumulation of amino acid metabolites in the mosquito. Number of pathways involved in maintaining redox state such as pentose phosphate pathway, citrate cycle and oxidative phosphorylation were found to be targeted by miR-124, miR-2944a-5p, miR-305-5p and miR-309. miR-309 was also identified to target nitrogen metabolic pathway essential for protein metabolism and detoxification of ammonia in the mosquito. Among the common targets, few mRNAs (AGAP004742, AGAP011551 and AGAP002564) coding for pyruvate carboxylase, protein transport protein SEC31 and aldolase respectively were previously found to get regulated post blood feeding and embryonic development in mosquito [45]–[46].

As in response to blood feed, successful infection by *Plasmodium* in mosquito also triggers multiple changes in metabolic, redox and immune related processes. Previous studies have shown that metabolic and redox state of mosquito can alter mosquito susceptibility to *Plasmodium* infection [47]–[48]. At iBF 42 h, up-regulated miRNAs namely miR-124 and miR-932 were identified to target pentose phosphate pathway and citrate cycle respectively. These targets were probably required for the controlled ROS generation in mosquito for optimal *Plasmodium* development and transmission to mammalian host. Further, we identified miR-137 targeting mRNAs playing role in TGF-beta signalling pathway, essential for mosquito immunity against *Plasmodium* parasite. One miRNA, miR-124 is of utmost importance as it was significantly down-regulated at BF 42 h whereas was up-regulated at iBF 42 h. It was identified to target 25 mRNAs lying in carbon metabolism, pentose phosphate pathway and protein processing in endoplasmic reticulum pathways. At iBF 5d, miR-281-3p was found to target mRNAs in TGF-beta signalling pathway. Glutathione metabolic pathway is a major redox pathway, whose genes were found to be targeted by miR-285. Further, many more metabolic pathways were identified to be targeted by miR-1174, miR-2944a-5p and miR-309 regulated at iBF 5d. Analysis of common targets revealed some important transcripts such as AGAP004192, AGAP000747 and AGAP011888 coding for heat shock protein, MAPKKK and exportin-5 respectively. Previous studies have shown their importance in pathogenesis of infectious diseases. Hosts are known to increase heat shock protein production in response to pathogen encounter [49]. Similarly, MAP protein kinases are a part of MAP Kinase signalling pathway playing role in anti-Plasmodium defence in mosquitoes [50]. Exportin -5 is involved in miRNA biogenesis pathway in different organisms. Such components of miRNA biogenesis pathway are known to get regulated in host cells to enhance the survival of infectious agents [51]–[52]. In our study, exportin-5 was found to be targeted by miR-1175-3p and miR-1891. miR-1175 was shown to get up-regulated post blood feeding whereas infection with *Plasmodium* parasite causes its down-regulation in the midgut of female mosquito [6]. A previous study has shown role of miR-1891 in longevity and fecundity of adult *Aedes albopictus* mosquitoes [53].

Parasites are known to alter host responses for their optimal growth and development. Infectious agents like HIV-1 regulate miRNA silencing pathway of the host during its replication [54]. Bacterial effector proteins are also known to suppress miRNA pathway to cause disease [55]. Uninfected blood is known to harbour number of factors resulting in differential expression of miRNAs. Differential expression of miRNAs upon parasite infection can either be a result of defence response of host against invading parasite or brought about by parasite itself to sustain its development. Therefore, whether the differential expression of miRNAs found in this study is attributed to host responses or are caused by invading parasite effector molecules needs further investigation.

This is the first comprehensive study to profile a large number of *An. stephensi* miRNAs at different time points post blood feeding and *Plasmodium* infection. Differentially expressed miRNA in BF and iBF were identified by small RNA sequencing. These were further validated by real time PCR. Novel miRNAs also showed regulated expression in BF and iBF. Many miRNAs identified were conserved in insects although few were identified for the first time in *Anopheles*. Target prediction revealed important mRNA candidates mapped to diverse biological pathways. miRNA:mRNA interaction network point towards complex regulation of processes post blood feeding and infection in the vector. Elucidation of regulated miRNA functions is an important challenge that will provide strong foundation for understanding parasite development and mosquito-parasite interactions.

## Supporting Information

Figure S1 **Figure S1 is a figure showing predicted secondary structure of novel miRNAs.** Mature miRNA were found only on one arm of pre-miRNA, represented by coloured sequences.
(TIF)

Table S1 **Table S1 is a table listing miRNAs identified containing mismatch with known miRNAs and with** ***An. stephensi*** **genome.**
(XLSX)

Table S2 **Table S2 is a table listing miRNAs regulated post blood feeding and infection and their targets predicted by RNA hybrid tool.**
(XLSX)
