# Supplementary figures and images for: Blood Feeding and Plasmodium Infection Alters the miRNome of Anopheles stephensi
Source: PLoS One. 2014 May 27;9(5):e98402. doi: 10.1371/journal.pone.0098402 (PMC4035286; doi:10.1371/journal.pone.0098402)

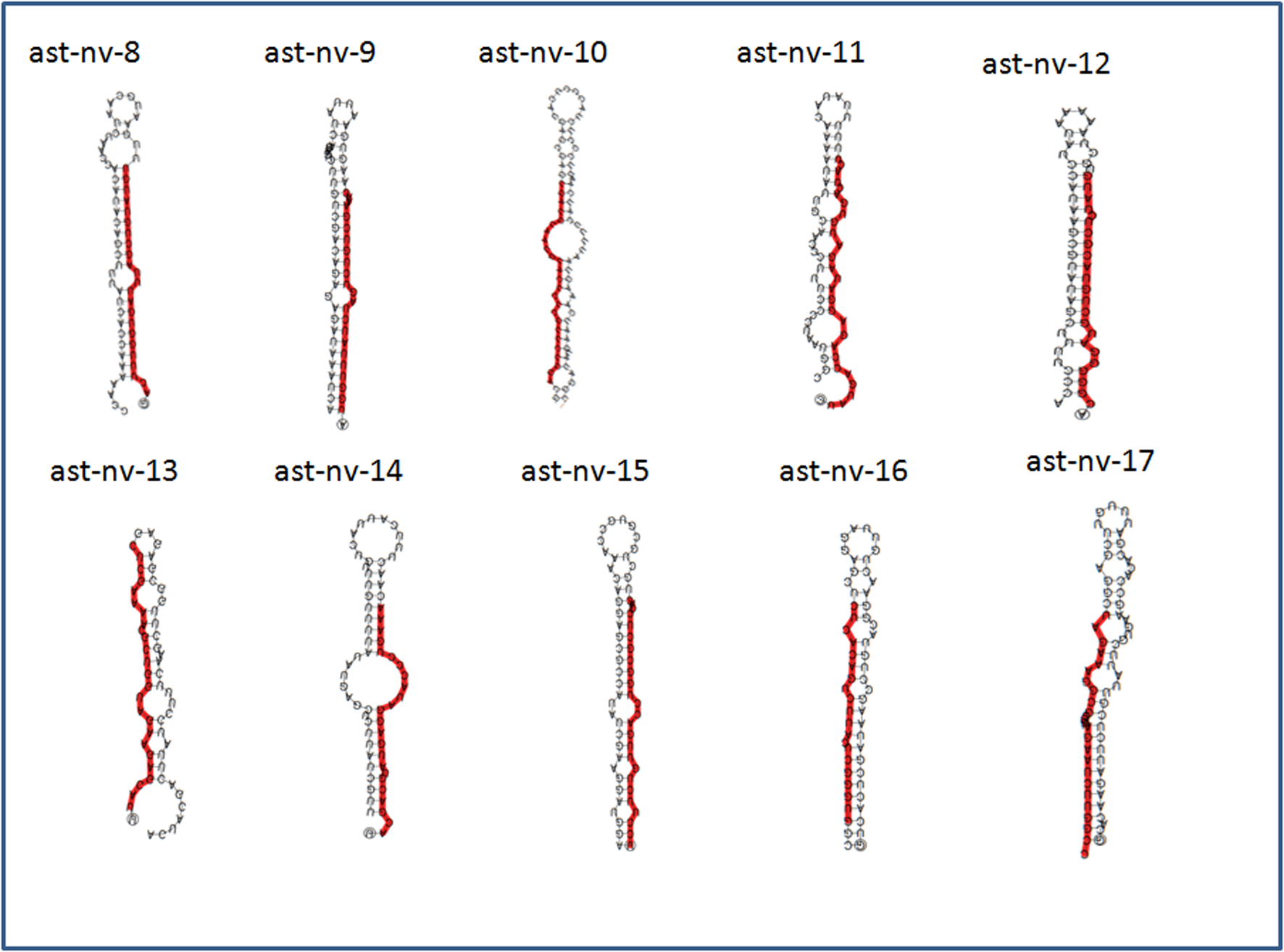

Supplement: Figure S1 — is a figure showing predicted secondary structure of novel miRNAs. Mature miRNA were found only on one arm of pre-miRNA, represented by coloured sequences. (TIF) [file pone.0098402.s001.tif]
